# Local processors: Active dendrites and artificial intelligence

**DOI:** 10.1016/j.crneur.2026.100169

**Published:** 2026-10-15

**Authors:** Yi Hu, Nicholas S. Glatz, Lucy M. Palmer

**Affiliations:** Florey Institute of Neuroscience and Mental Health, Florey Department of Neuroscience and Mental Health, University of Melbourne, Parkville, VIC, Australia

## Abstract

Neuroscience and artificial intelligence have been closely connected for over a century, with each field influencing the other. Modern artificial intelligence systems rely almost exclusively on the point-neuron model, where inputs are linearly summed and passed through a global activation function. Although highly successful, this architecture differs substantially from neural computation that occurs in the brain, and current leading models require massive infrastructure consuming megawatts of power, which contrasts to the brain's remarkable efficiency. One underexplored source of biologically inspired computational principles lies in the dendrites of pyramidal neurons. This review examines the electrical properties of active dendrites and how they could inform the next generation of artificial intelligence architectures. We describe how dendrites can function as semi-independent computational subunits, generating local nonlinear events that implement coincidence detection, signal amplification, and logical operations within a single neuron. We discuss how the structural segregation of different dendritic compartments provides a biologically grounded alternative to global error backpropagation through local credit assignment. We further examine how activity-dependent spine growth and pruning support continual learning without catastrophic forgetting, and how the spatiotemporal selectivity of dendritic integration gives rise to sparse, energy-efficient representations. Together, dendritic computation represents a potential blueprint for artificial intelligence architectures that are adaptive, context-aware, and energetically frugal.

## Highlights

• This manuscript explores the electrical properties of active dendrites and how they could inform the next generation of artificial intelligence architectures.
• Dendrites can function as semi-independent computational subunits, generating local nonlinear events that implement coincidence detection, signal amplification, and logical operations within a single neuron.
• The structural segregation of different dendritic compartments provides a biologically grounded alternative to global error backpropagation through local credit assignment.
• Activity-dependent spine growth and pruning supports continual learning without catastrophic forgetting seen in artificial network models.
• Dendritic sparse, selective coding can reduce redundant computation and enable energy-efficient, event-driven processing in specialized hardware.
• We highlight that the dendrites of pyramidal neurons may act as a potential blueprint for artificial intelligence architectures that are adaptive, context-aware and energetically frugal.

## Graphical abstract

**Figure:**
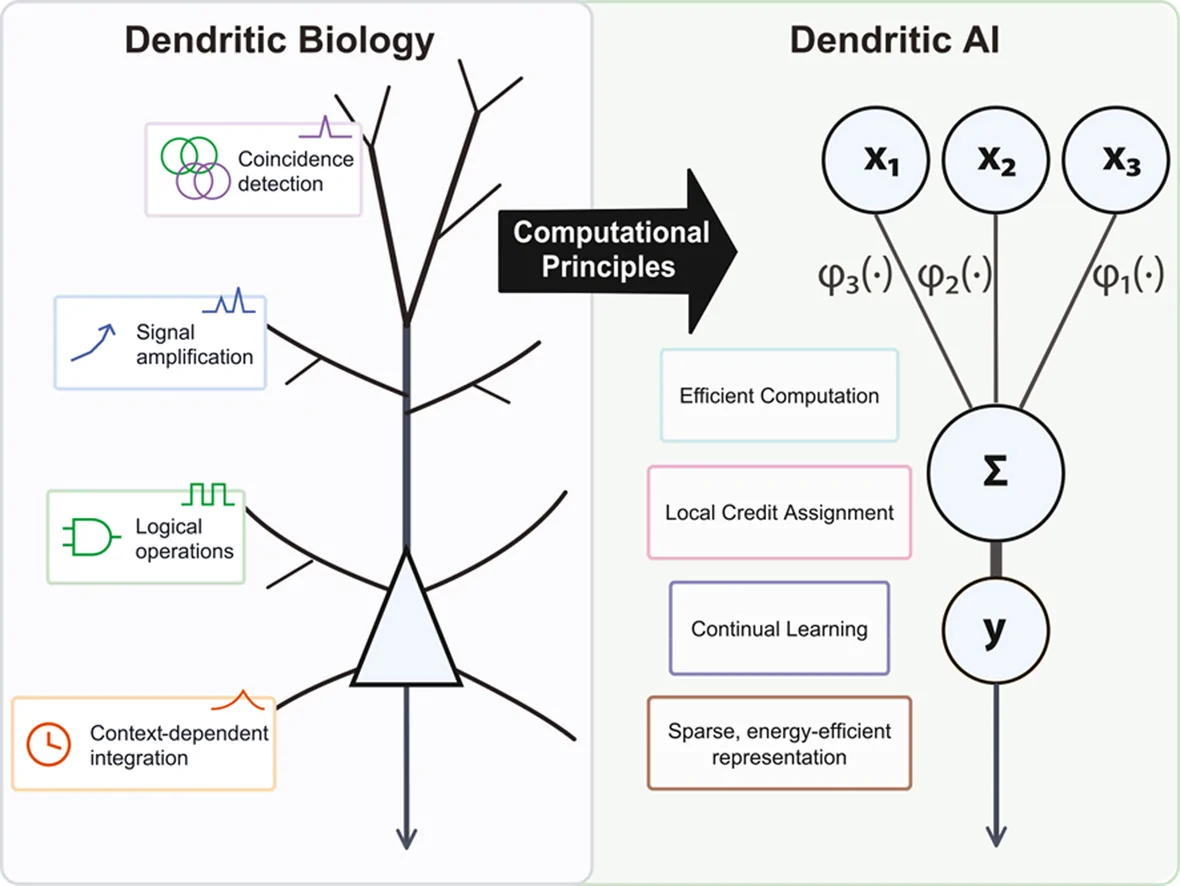

## 1 Introduction

Since the emergence of machine learning in the mid-20th century, the fields of neuroscience and artificial intelligence have been intertwined. Early artificial neural networks were directly inspired by early neuroscience principles. For example, the perceptron was a simplified model of a neuron, and Hopfield networks were based on a physicist's interpretation of the neurobiology of associative memory. Even advanced contemporary architectures, such as the self-attention mechanisms in transformers, trace their conceptual lineage back to the biological study of attention. As new principles of biological neural computation continue to be developed and refined in neuroscience, they may provide valuable inspiration for the development of artificial intelligence models.

Modern artificial intelligence systems rely almost exclusively on an extremely simplified model of neural computation, the point-neuron model (McCulloch and Pitts, 1943). In this framework, inputs are weighted and linearly summed, then passed through a continuous activation function (such as ReLU or Sigmoid) to produce a numerical output. Although this architecture has proven computationally tractable and highly successful, it differs substantially from the computation that occurs in the brain. More advanced deep learning models, such as large language models, require substantial infrastructure, relying on massive GPU clusters and consuming megawatts of power during both training and inference. While the brain is metabolically expensive relative to its size, responsible for approximately 20% of the body's energy consumption, despite comprising only 2% of its mass, it is able to perform remarkably complex computations on a limited energy budget, consuming approximately 20 W of power (Attwell and Laughlin, 2001; Raichle and Mintun, 2006). These differences in energy consumption suggest that important computational principles underlying the brain's energy efficiency remain largely underutilized in current artificial intelligence architectures. One potential source of such principles lies in pyramidal neurons, the principle excitatory neurons that reside in the cortex, hippocampus and subiculum. In particular, the dendritic tree of pyramidal neurons expands the computational capabilities of individual neurons beyond those captured by point-neuron models. As a result, dendrites have attracted significant interest from those attempting to understand and build structures that can perform complex computations. We do not propose dendritic mechanisms as universally optimal solutions to the challenges faced by AI researchers; rather, we will highlight the impressive computational capabilities that emerge from distinct, biophysical dendritic mechanisms, examine the computational principles emerging from these capabilities, and consider how these principles may inspire solutions to challenges in AI that parallel specific problems addressed by biological neural systems, including credit assignment, continual learning, and energy efficiency.

The goal of this review is not to provide an exhaustive survey of the literature on dendrites, but rather to highlight specific principles and functions of dendrites which may be relevant to artificial intelligence models. Focusing on pyramidal neurons in cortex and hippocampus in the adult human and rodent brain, this review will also focus on the electrical, not biochemical, properties of dendrites. While these properties can be difficult to isolate in biological neurons, as an abstraction, they are more readily translated into artificial intelligence models.

## 2 Beyond point-neurons: compartmentalized and nonlinear dendritic computation

The computational capabilities of the neuron are not static; they are dynamically shaped by experience. This capacity for dynamic neuronal adaptation is a fundamental driver of learning and memory, and results in changes that occur across a range of time scales, from seconds to weeks, and in some cases, can persist for a lifetime. Dendrites play a key role in these dynamic processes. By integrating incoming signals and supporting synaptic plasticity, dendrites provide critical structural and functional neuronal infrastructure. Understanding the brain's remarkable computational capabilities therefore requires considering the full morphology of neurons, including dendrites.

### 2.1 Structural segregation and functional compartments in pyramidal neurons

Pyramidal neurons exhibit a stereotyped polarized morphology consisting of two distinct dendritic regions. The soma (cell body) is shaped like a rounded pyramid, from which an apical dendritic trunk extends and bifurcates into a sprawling tree. In addition, several shorter basal dendrites also emerge from the soma and typically spread laterally (Fig. 1A). These two dendritic compartments differ in their received inputs, integrative properties, and morphology (Benavides-Piccione et al., 2024; Jiang et al., 2020; Thieken et al., 2016; Petreanu et al., 2009; Wimmer et al., 2010), generating functional compartmentalization within the neuron. A substantial body of work has demonstrated that dendritic compartmentalization plays an important role in driving the computations performed by pyramidal neurons (for a review, see (Koch and Segev, 2000), (Major et al., 2013)). This dendritic organization has been suggested to drive association processing in cortical circuits, with basal dendrites primarily receiving feed-forward sensory input, while apical dendrites primarily receive feedback signals conveying contextual or higher-order information from other cortical and subcortical regions (Rubio-Garrido et al., 2009; Manita et al., 2015; Larkum et al., 2004; Galloni et al., 2022; Shen et al., 2022). The interaction between these streams of input provides a mechanism through which internally processed information can amplify selected environmental input, allowing the neuron to integrate external input with internal feedback signals (Silverstein et al., 2026; Adeel et al., 2023; Larkum, 2022; Phillips, 2023).

**Fig. 1:**
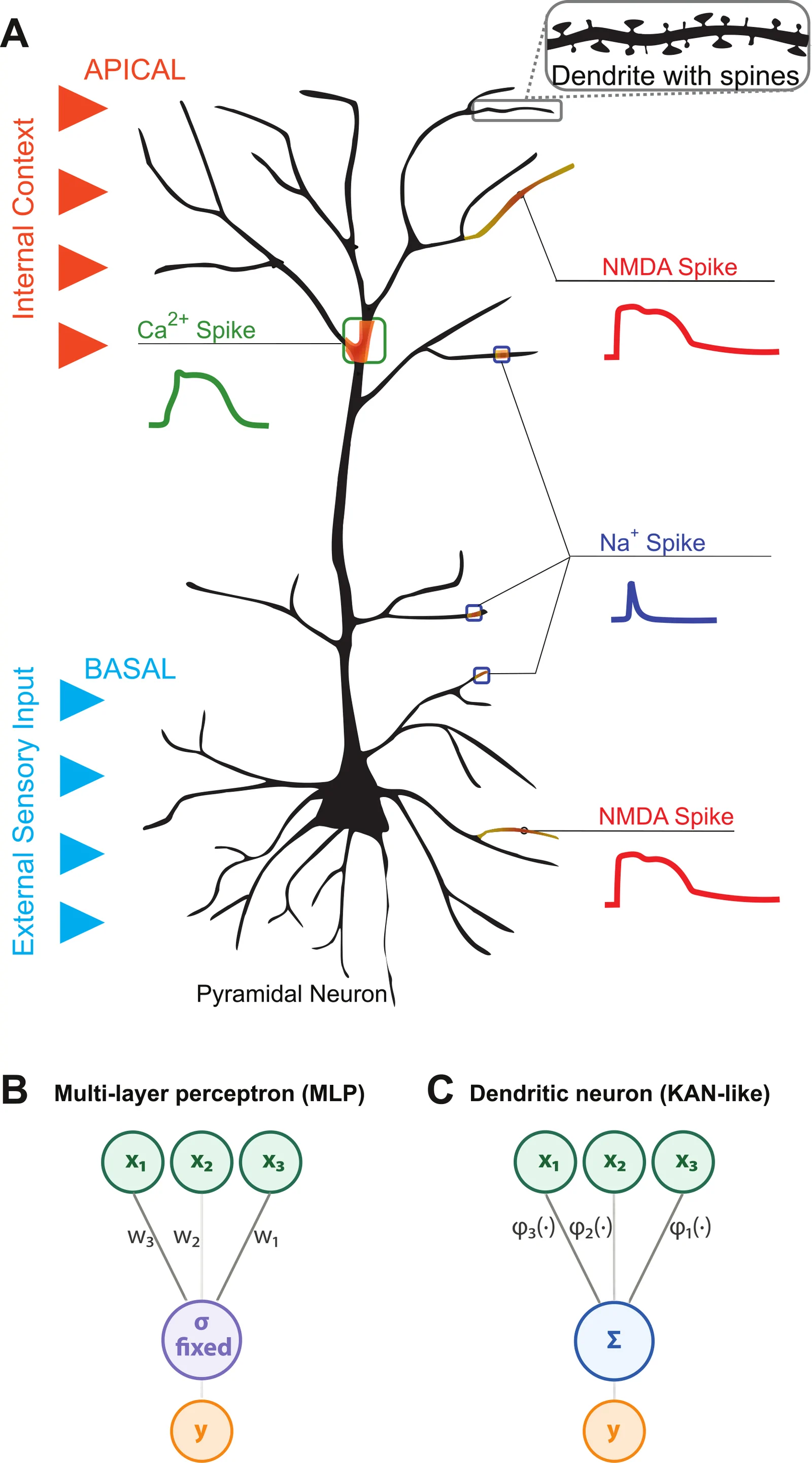
**Structural segregation and dendritic spikes in pyramidal neurons. (A)** Schematic of a cortical pyramidal neuron illustrating how feedforward input (blue) targets basal dendritic compartments and feedback input (orange) targets apical dendritic compartments. Dendritic location of dendritic spike generation: NMDA spikes (AND gate, coincidence detection), Ca^2+^ spikes (global burst from apical hotspot), and Na^+^ spikes (local filter). **(B)** Example of multi-layer perceptron (MLP) neuron Weighted (w_n_) input (x_n_) is transformed by a sigmoid activation function (σ) at the node. a fixed sigmoid activation function (σ) at the node before generating output (y). (**C)** Example of dendritic neuron (KAN-like): input (x_n_) is transformed by a learnable activation function (Φ_n_) at the connections (edges), before being summed (Σ) at the node and generating output (y).

Although the functional role of pyramidal neurons has been the subject of extensive investigation, the appropriate level of granularity for describing dendritic computation remains an active area of debate. This question has often been approached by examining the minimum size of a neuron's functional compartment required to explain the input-output behavior of the neuron (Häusser and Mel, 2003). Several models have been proposed, from entire dendritic regions to individual dendritic spines (Fig. 1A). This variability in the degree of granularity is, in part, due to the different questions being asked and the level of analysis being considered.

Increasing the granularity of dendritic compartmentalization can, in principle, increase computational capacity by allowing a single neuron to perform multiple non-linear operations in parallel. However, too many dendritic compartments may go beyond the resolution that is functionally relevant for understanding neural network dynamics. Consequently, the extent, feasibility and functional importance of dendritic compartmental segregation remain a subject of ongoing discussion (Häusser and Mel, 2003; Branco and Häusser, 2010; Yuste, 2013). However, it is clear that dendritic compartmentalization allows multiple computations to occur in parallel, substantially expanding the integrative capacity within a single neuron. Here, individual dendritic branches will be considered as the dendrite's functional compartment, with the caveat that other neural structures, such as dendritic spines, may serve as smaller compartments.

### 2.2 Local and global activation functions

Traditionally, dendrites were viewed as passive cable-like structures (Rall, 1964). In this classical view, synaptic inputs arriving along the dendritic tree were thought to linearly integrate as they propagated toward the soma. Since electrical signals attenuate with distance, inputs located far from the axonal site of action potential initiation were assumed to exert a weaker influence on action potential generation. Therefore, classically, dendrites were posited to primarily serve as passive conduits that filtered and summed inputs. However, it is now known that the dendrites of pyramidal neurons can transform synaptic input into voltage signals that exceed the linear sum of the individual synaptic input (Schiller et al., 2000; Gasparini and Magee, 2006). Known as dendritic spikes or plateau potentials, these supralinear voltage events are generated according to the spatial distribution and timing of synaptic activity as well as the ion-channel driving the conductance (Major et al., 2013; Larkum and Nevian, 2008; Johnston and Narayanan, 2008). Dendritic spikes play an important role in neuronal excitability, leading to the generation of action potentials in pyramidal neurons (Grienberger et al., 2014; Larkum et al., 2009; Palmer et al., 2014). Human cortical dendrites additionally generate a distinct, calcium-mediated dendritic action potential capable of implementing logical operations, including exclusive-OR (XOR), within a single dendritic branch (Gidon et al., 2020), whose computation classically requires a hidden layer in a multilayer perceptron.

The properties of dendritic spikes vary depending on the underlying ionic conductance that generates them, leading to the generation of dendritic spikes that have differing temporal and spatial scales, including NMDA spikes, Ca^2+^ spikes and Na ^+^ spikes. Dendritic spikes that are driven by the activation of N-methyl-D-aspartate (NMDA) receptor-channels are called NMDA-spikes (Larkum and Nevian, 2008; Schiller and Schiller, 2001; Yang et al., 2014a). Requiring both glutamate binding and sufficient local depolarization to remove a Mg^2+^ block form the channel pore (Nowak et al., 1984; Kampa et al., 2004), NMDA-spikes can therefore act as a coincidence detection mechanism in both rodent and human neurons (Testa-Silva et al., 2022). Following this, NMDA-spikes can powerfully transform and nonlinearly amplify relevant signals, effectively implementing a nuanced form of an AND function (Fig. 1A; 24). A single NMDA spike is locally restricted (Palmer et al., 2014; Polsky et al., 2004), with minimal spread along a dendritic branch (30 μm spread), and leads to a relatively long period of depolarization ranging between 50 and 100ms (Schiller et al., 2000; Rhodes, 2006), Dendritic spikes can also be generated by voltage-gated calcium channels, termed ‘Ca^2+^ spikes’ (Larkum et al., 1999). Generated in the apical dendrite, Ca^2+^ spikes can spread globally and cause a relatively sustained depolarization (10s to 100s of ms) (Magó et al., 2021). Upon reaching the soma, they can strongly enhance a neuron's regular firing properties by generating a burst of action potentials (Larkum et al., 1999). In contrast, dendritic spikes mediated by voltage-gated sodium channels, referred to as ‘Na^+^ spikes’, are typically smaller, rapidly rising and decaying in milliseconds and are spatially restricted, allowing for more local computation (Katz et al., 2009). Since there is a relatively low but widespread distribution of voltage-gated Na^+^ channels throughout the dendritic membrane (Lorincz and Nusser, 2010), Na^+^ spikes can occur throughout the dendritic tree (Ariav et al., 2003; Golding and Spruston, 1998). Na^+^ spikes can boost signal that exceeds a threshold locally without necessarily propagating to the soma, enabling semi-independent computations within dendritic compartments in both rodents and humans (Fig. 1A; (Golding and Spruston, 1998), (Gasparini et al., 2004), (Gooch et al., 2022)).

Importantly, dendritic spikes do not occur in isolation. Dendritic spikes can also interact, giving rise to nested computations within the dendritic tree. For example, multiple NMDA-spikes can contribute to the generation of a Ca^2+^ spike, which in turn can generate somatic action potentials (Palmer et al., 2014; Xu et al., 2012). Through these interacting nonlinear dendritic spikes, dendrites provide neurons with a powerful cellular mechanism that can efficiently transform synaptic input into action potential output.

In addition to dendritic spikes, further computational possibilities are created by back-propagating action potentials (bAPs). bAPs are generated in and travel from the axonal initiation site back into the dendritic tree following somatic firing. Their amplitude generally attenuates with distance along the dendritic arbor, propagating on a millisecond timescale (Stuart and Sakmann, 1994; Waters et al., 2005). In distal dendrites, bAPs can interact with coincident synaptic input to trigger regenerative Ca2+ spikes, which in turn can propagate toward the soma and promote burst firing. Consequently, bAPs provide a mechanism by which somatic output can interact with distal dendritic input, across the neuron, enabling nonlinear integration of information at different locations and times in the neuron. This is particularly powerful given the preferential segregation of top down and bottom-up information streams arriving to the apical and basal dendrites. Feedforward input can arrive at the basal dendrites and contribute to somatic firing, with the resulting bAP interacting with coincident feedback inputs at the distal apical dendrites. Such interactions can promote burst firing, allowing individual neurons to form associations between sensory and contextual information.

Together with their passive filtering properties, dendritic spikes equip dendrites with the means to perform coincidence detection, filter out noisy input and amplify distinct signals. Recent developments in artificial intelligence suggest that implementing such non-linear transformations at the site of the connection between neurons, or nodes, could enhance future models. A striking parallel to dendritic computation can be seen in the newly proposed Kolmogorov-Arnold Networks (KANs). In contrast to multilayer perceptrons which apply fixed activation functions at neurons (nodes; Fig. 1B), KANs place learnable non-linear activation functions at the connections (edges; Fig. 1C) (Liu et al., 2024a). In this framework, the “connection” between two nodes is not simply a static scalar weight, but a dynamic, trainable non-linear transformation function. This design closely parallels biological dendritic computation, in which synaptic inputs are transformed nonlinearly within dendritic compartments before influencing somatic output. KANs have achieved higher accuracy and interpretability with fewer parameters than multilayer perceptrons, illustrating the potential benefits of assigning greater computational power to the connections between units. Inspired by dendritic physiology, future artificial intelligence architectures may therefore benefit from exploring non-linear edge transformations that resemble the dynamics of dendritic spikes.

## 3 Localizing credit using synaptic plasticity and dendritic integration

A persistent challenge in artificial intelligence is the credit assignment problem, where a network must appropriately alter internal parameters to adjust to output errors. The most common approach to tackle this problem is error-backpropagation, in which an error signal is propagated backwards through the layers of the network to update trainable parameters (Fig. 2A). Although mathematically and conceptually elegant, backpropagation is computationally demanding because it requires a new error gradient to be calculated for the entire network with each iteration. It is also considered biologically implausible, as it requires symmetric feedback pathways and transport of error signals across layers (Lillicrap et al., 2020; Hinton and Sejnowski, 1986; Bengio et al., 2015). Implementing global error propagation is limited in neuromorphic hardware, where specialized communication channels limit the scalability of centralized learning rules in distributed systems (Grossberg, 1987).

**Fig. 2:**
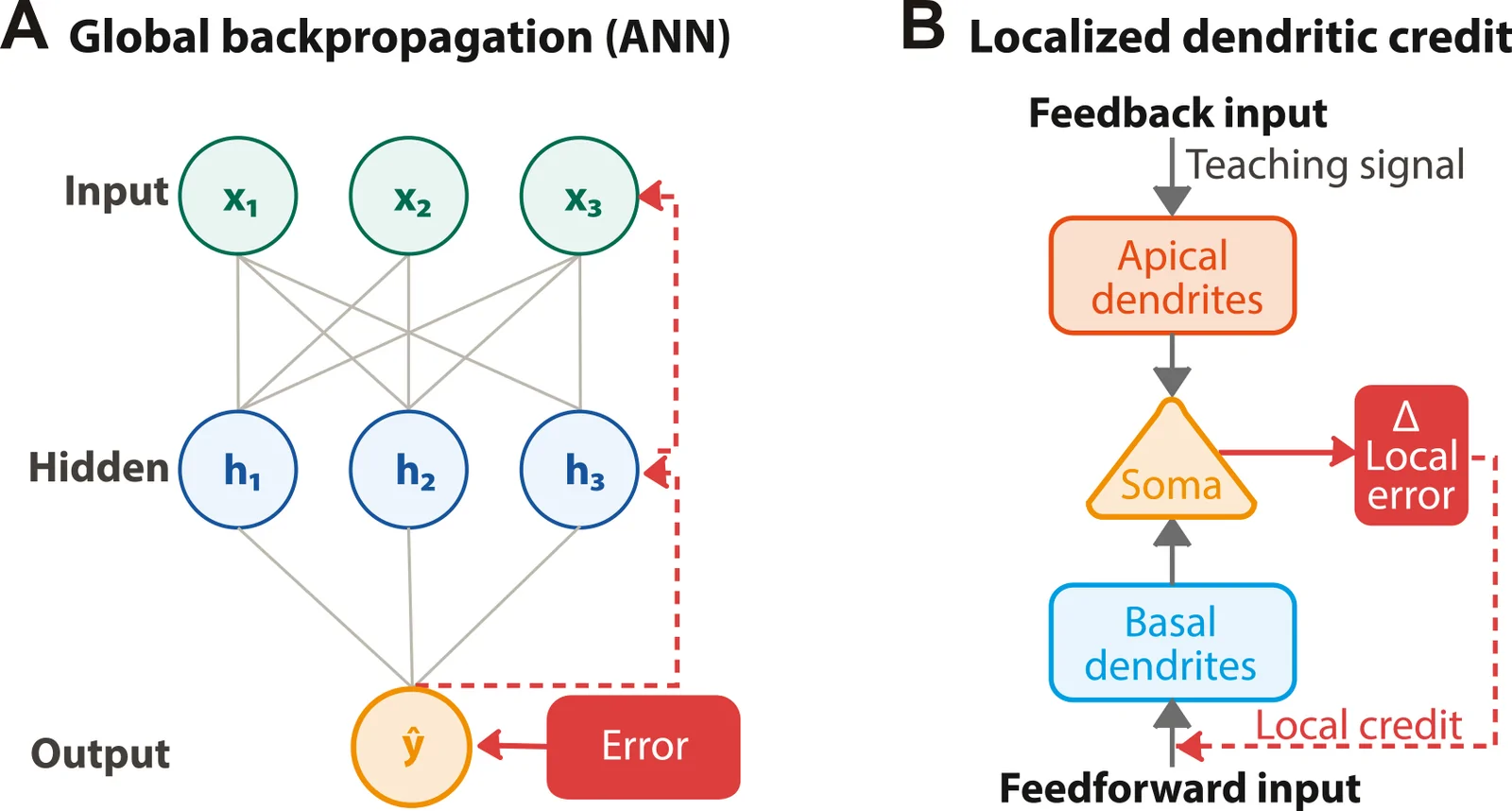
**Credit assignment: global backpropagation vs. local dendritic error signaling. (A)** The standard ANN approach: an error signal propagates backward through every layer, requiring symmetric feedback pathways and an expensive full gradient recalculation for each pass. Error signal, dashed line. **(B)** Local dendritic credit solution: the mismatch (Δ) between the top-down apical teaching signal and the bottom-up basal input generates a local error, which drives plasticity at individual synapses (dashed line), and hence no global error pipeline is required.

Beyond biological plausibility, backpropagation carries concrete engineering costs: conventional implementations require intermediate activations and gradients to be retained for the backward pass, while the sequential dependence between forward and backward computations introduces synchronization and communication overhead. While in conventional digital architectures with abundant shared memory and high-bandwidth communication, backpropagation remains highly effective, these costs can become particularly restrictive in spatially distributed and neuromorphic hardware, where memory and communication resources are constrained (Renner et al., 2024; Mostafa et al., 2018; Van Doremaele et al., 2024). Local learning rules offer a potential alternative by allowing synaptic updates to be computed from locally available feedforward and feedback signals, reducing the need for network-wide storage, synchronization, and error propagation.

Dendritic computation provides a biologically grounded framework for local credit assignment. In pyramidal neurons, the segregation of input pathways across different dendritic compartments provides a potential substrate for local credit assignment (Fig. 2B (Francioni et al., 2026);). Biologically inspired models have demonstrated how an error signal sent to the apical dendritic compartment could function as a local teaching signal by modulating the efficacy of relevant synapses (Urbanczik and Senn, 2014; Sacramento et al., 2018; Payeur et al., 2021). This mechanism approximates backpropagation locally, with each neuron minimizing its own prediction error in coordination with surrounding units. In cortical pyramidal neurons, this computational capacity is further supported by a general segregation of feedforward sensory inputs onto basal dendrites, and contextual, or feedback, inputs onto apical dendrites (Rubio-Garrido et al., 2009; Manita et al., 2015; Larkum et al., 2004; Galloni et al., 2022; Shen et al., 2022). This organization enables local computations and learning through the contextual modulation of sensory input locally. In biological systems, synaptic plasticity and dynamic dendritic integration provides a flexible substrate to drive local associative learning (for a review, see (Nicoll, 2017)). Artificial intelligence systems could adopt local learning rules that integrate feedforward input and feedback error within individual units, reducing the need for global error propagation (Richards and Lillicrap, 2019; Meulemans et al., 2022). Localized learning would allow parameters to be updated semi-independently rather than requiring a complete update of the whole neuron or network. Distributed representations could, in turn, allow networks to generalize and adapt to new environments more effectively than conventional computational networks, which often compress diverse information into unified representations (Hinton, 1984). Furthermore, segregation of different information streams in pyramidal neurons would enable the creation of modular sub-networks that separately encode complimentary information pathways, expanding the network's integrative capacity (Sacramento et al., 2018; Richards and Lillicrap, 2019). The advantage of segregated information streams can already be seen in successful computational network architectures employing multiple processing pathways and attention mechanisms to achieve partially segregated information streams (Vaswani et al., 2017; Dosovitskiy, 2020). This decentralized approach could also reduce communication overhead, offering a scalable path for learning in hardware-constrained and distributed environments (Sacramento et al., 2018; Payeur et al., 2021). Taken together, dendritic computation and local synaptic plasticity may provide an alternative route to addressing the credit assignment problem while expanding the computational flexibility of artificial neural networks.

## 4 Dendritically driven structural adaptability

Most artificial neural networks operate with fixed architectures, where learning occurs through changes in synaptic weights, while the network topology remains static. In contrast, biological neural circuits are continuously reshaped by experience (Xu et al., 2009; Yang et al., 2009; Chen et al., 2015). This plays a critical role in allowing organisms to learn throughout their lives while maintaining valuable functions. While there are morphological changes to the overall dendritic structure during development, the morphology of dendritic branches is particularly stable in the adult brain (Holtmaat and Svoboda, 2009; Maravall et al., 2004). Instead, morphological changes occur at the site of synaptic input onto dendrites, dendritic spines. Spines can grow or shrink, appear or disappear *in vivo* (Fig. 3A) (Attardo et al., 2015; Pfeiffer et al., 2018; Zuo et al., 2005). For example, when animals learn new motor skills, there is an increase both the formation and elimination of spines in the motor cortex (Xu et al., 2009; Yang et al., 2009). Newly formed spines can appear in spatial clusters (Hedrick et al., 2022), which may link related inputs within the same spatial location (Fig. 3B) (Yuste, 2011). Such branch-specific changes can reduce interference between unrelated memories by enabling local consolidation within dendritic compartments (Fu et al., 2012; Yang et al., 2014b). These biological processes may be adopted by artificial systems to allow more dynamic learning.

**Fig. 3:**
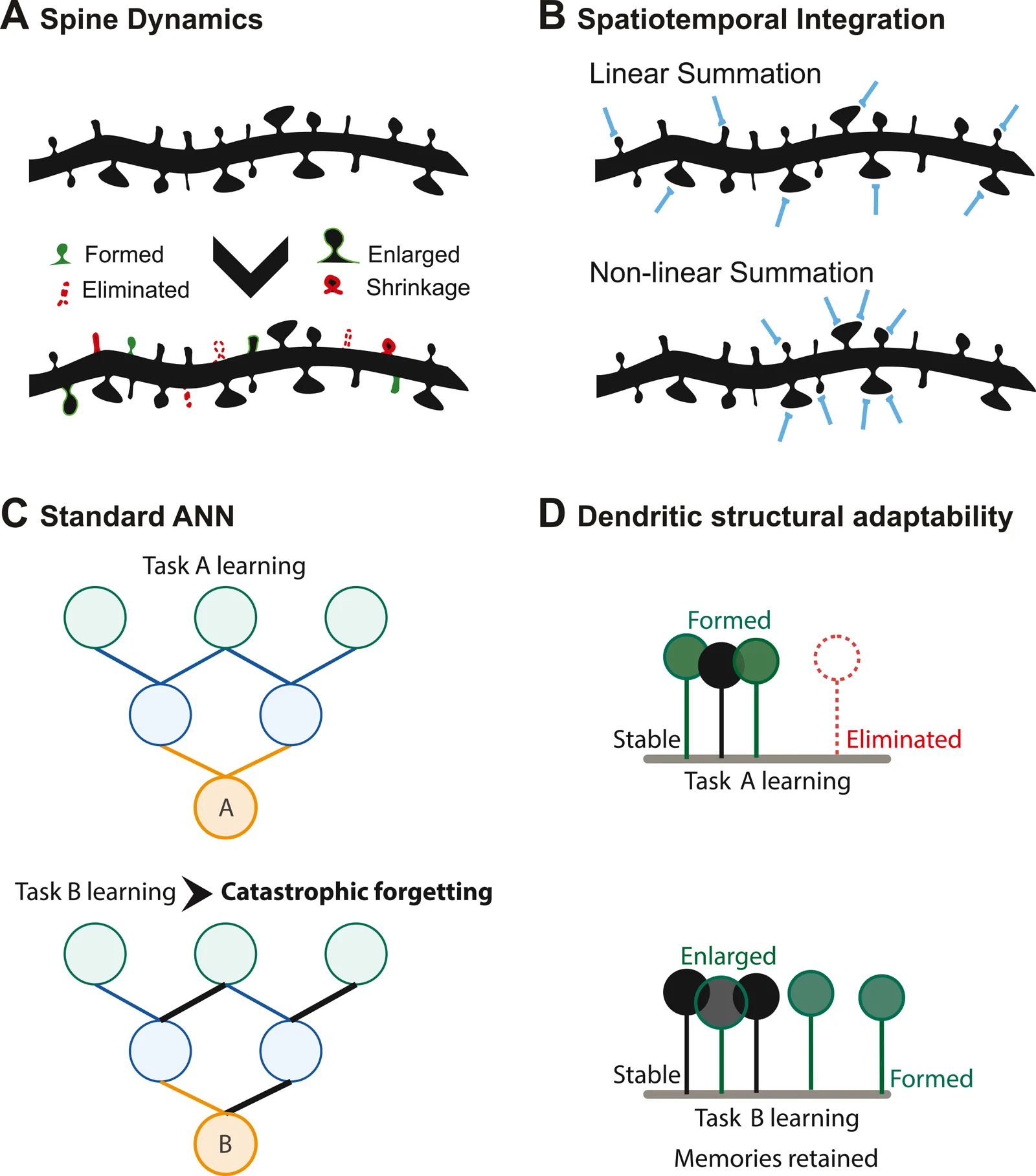
**Structural adaptability. (A)** Spine dynamics and **(B)** Spatiotemporal integration of synaptic input. Blue axons indicate spines with inputs. **(C)** Training Task B in a static ANN can directly overwrite the weight configuration learned for Task A, causing catastrophic forgetting. Overwritten weights, thick (black) line. Stable weights, thin line. **(D)** Schematic examples of how biological dendritic structural adaptability prevents catastrophic forgetting. (top) Task A is encoded in a functional cluster of dendritic spines involving spine formation (green) and elimination (red). (bottom) Learning of Task B results in the formation of new (green) and enlarged (green outline) spines. Homeostatic plasticity maintains total activity at a set point, preserving both memories.

A major limitation in modern Artificial Neural Networks (ANNs) is catastrophic forgetting, where the global weight updates required to learn new tasks often overwrite the parameters used for previously acquired knowledge (McCloskey and Cohen, 1989; Goodfellow et al., 2013) (Fig. 3C). This challenge is one faced by all adaptive networks, which must preserve previously acquired knowledge, while remaining sufficiently plastic to learn new information. Several methods have been developed to overcome catastrophic forgetting and address this trade-off in ANNs. For example, Elastic Weight Consolidation (EWC) and null-space learning strategies constrain parameter updates to stabilize previously acquired representations, progressively restricting changes to previously important parameters (Loke et al., 2025; Schwab et al., 2019). Another approach, task-specific modularity, adds additional capacity and utilises task-specific routing to prevent existing parameters from being overwritten (Suteu and Serban, 2025). Dendritic compartmentalization offers a framework in which stability and plasticity can be spatially segregated within individual neurons, allowing computation, plasticity and memory to be co-localized within semi-independent computational units. In contrast to the global constraints on plasticity imposed by methods such as EWC and null-space learning, dendritic compartmentalization allows established representations to be preserved within stable spines, while new information is incorporated at other synaptic sites. Compared to specialized gating modules, dendritic structural plasticity could provide a finer-grained mechanism for allocating new learning capacity, allowing new representations to be incorporated without the coordinating and interference costs required by explicit task boundary signals or task dedicated network modules.

Adaptive or self-organizing artificial intelligence architectures could, like biological systems, dynamically modify their connectivity, forming new computational pathways while pruning inefficient ones (Fig. 3D). Such strategies resemble evolutionally and neuro-inspired algorithms that jointly evolve network topology and synaptic weights to balance flexibility with efficiency (Stanley et al., 2019). Recent artificial intelligence implementations, such as Dynamic Graph Neural Networks and Liquid Neural Networks, have begun to explore architectures that move beyond static architectures (Chen et al., 2025a; Hasani et al., 2021; Peng et al., 2020). However, in many current implementations, the underlying connectivity remains fixed over time, even if internal activations are dynamic.

Future artificial models could incorporate activity-dependent structural plasticity that deletes connections, or edges, and creates new connections based on activity, enabling adaptation to the network's architecture. Dynamically modifying network topology remains technically challenging in both software and neuromorphic hardware, and will require new approaches to efficiently implement structural plasticity before its potential benefits can be fully realised. However, innovation in implementing these architectures could yield significant benefits, enabling networks to gradually adapt their topology as learning progresses and may overcome catastrophic forgetting in a similar fashion to biological neural networks. Therefore, artificial intelligence architectures inspired by known synaptic and dendritic computation could harness the strengths of biological neural networks: being adaptive yet stable, self-organizing yet computationally efficient.

## 5 Learning the structure of time

Dendrites are spatiotemporal integrators, performing computations based on the spatial and temporal patterns of synaptic input. Specifically, the spatial and temporal proximity of synaptic input onto a dendritic tree determines how individual inputs are integrated; synaptic input that occurs separately in time and space typically sums linearly (Fig. 3B) (Polsky et al., 2004). However clustered input, that is, synaptic input which is functionally active close in time (within milliseconds) and space (within microns), can potentiate synaptic strength at the input site (Weber et al., 2016) and lead to the generation of dendritic spikes (Palmer et al., 2014; Polsky et al., 2009; Major et al., 2008; Losonczy and Magee, 2006; Takahashi et al., 2012). Clustered input has been shown to occur *in vivo*, where task-related synaptic activity was spatially clustered within individual dendritic branches after learning (Fu et al., 2012; Kerlin et al., 2019).

This tuning of dendrites to specific spatiotemporal patterns of synaptic input expands the computational capability of individual neurons, allowing a single branch to become specifically tuned to recurring temporal patterns. Based on this, a model which integrated dendritic temporal coincidence signaling enabled neurons to detect and predict complex higher-order temporal patterns, leading to significant improvements in the overall computational efficiency of the model (Hawkins and Ahmad, 2016). In contrast, while transformers are very successful at maintaining temporal dependencies within their context windows, the quadratic scaling of attention makes long temporal contexts very costly (Fichtl et al., 2025).

To enable local computation to occur within a single input layer, future models could use multi-compartment nodes with non-linear “dendritic” subunits. This would allow nodes to extract temporal dependencies directly without requiring explicit recurrent network-level connections or being limited by quadratic complexity, as is common in current leading artificial intelligence architectures. The possibilities and feasibility of such dendritic-inspired subunits for artificial intelligence can be seen in frameworks such as Hierarchical Temporal Memory (Hawkins and Ahmad, 2016), which simulated the mammalian neocortex by employing active dendritic segments as sequence-memory units that generate predictions of upcoming states. More recently, dendritic neuron models have integrated localized non-linear processing to perform complex logic within single-cell structures (Pagkalos et al., 2023). Another example can be seen with Liquid Neural Networks, which exploit dynamic, input-dependent time constants to enable continuous real-time adaptation (Hasani et al., 2021). These frameworks demonstrate the exciting possibilities held by models inspired by biological dendrites.

## 6 Dendritic sparse coding: efficient computation through selective activation

Sparse coding describes a representational strategy in which a given input or feature is represented by the strong activation of a small, specialized subset of neurons, while most neurons remain inactive (Barth and Poulet, 2012). Since information is typically carried by specific combinations of active neurons, sparse coding enables a vast number of unique representations within a neural circuit while maintaining high energy efficiency (Fig. 4). While traditionally described at the level of neural populations, sparse coding can also be driven within a single neuron through dendritic mechanisms. Dendritic spikes act as selective, non-linear filters that allow a single neuron to encode multiple distinct features or contexts, effectively functioning as a single high-dimensional processing unit (London and Häusser, 2005). At the network level, such localized and transient dendritic activity supports the efficient representation of complex sequences through the selective engagement of select dendritic branches providing a powerful and efficient substrate for brain functions (Olshausen and Field, 2004).

**Fig. 4:**
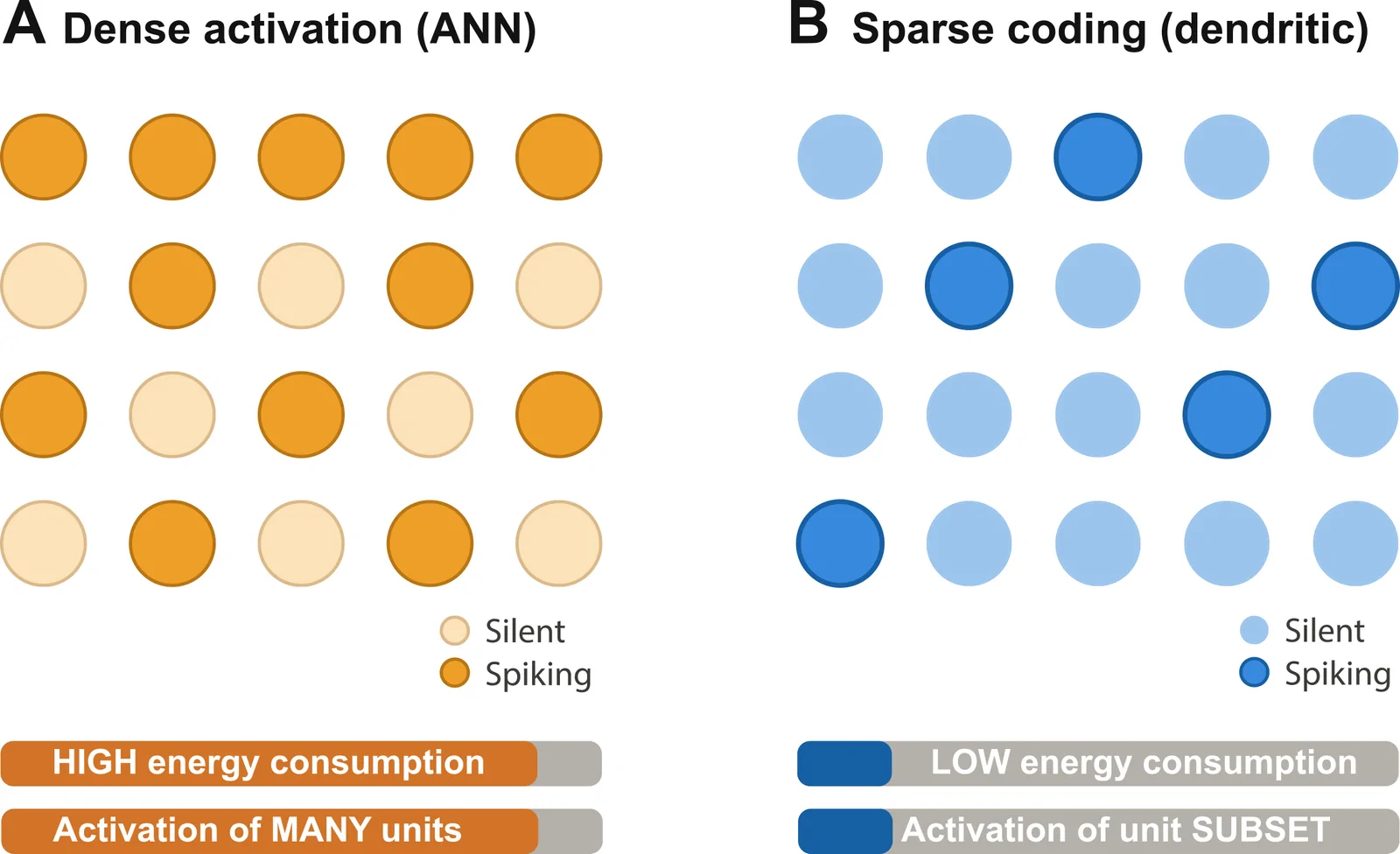
**Dense activation vs. sparse coding. (A)** Most neurons fire together in a standard ANN, with high energy consumption. **(B)** Sparse coding strategy: representations are made with a subset of specialized units firing together. The code is event-driven, energetically frugal, and the identity of the active subset uniquely identifies the stimulus. An exponential number of patterns can be stored without interference because their active ensembles are distinct.

Dendritic spikes have meaningful metabolic costs, as all of these active processes require energy to power ion-channel gating and Na^+^/K^+^-ATPase pump activity. However, by providing greater computational capacity to individual neurons and supporting sparse coding, dendritic mechanisms can reduce the number of neurons that need to be active to represent distinct features or computations. This is particularly relevant to the brain's relatively high energetic efficiency in performing complex computations, as synaptic transmission has been shown to be the single largest metabolic cost in the brain (Attwell and Laughlin, 2001; Tomasi et al., 2013). Consequently, dendritically driven sparse coding promises energy savings by reducing overall neural activity while retaining complex computational capabilities. It should be noted that these savings are not automatically portable to silicone. On conventional GPUs, dense matrix operations mean sparsity may have a limited energetic benefit. Architectures wishing to take advantage of the energetic savings promised by dendritic sparse coding will likely require dedicated event-driven or neuromorphic engineering rather than sparse activation patterns simulated within conventional architectures.

Current state-of-the-art deep learning models consume substantial energy during training and inference (Strubell et al., 2019; Thompson et al., 2020; Desislavov et al., 2023). Sparse, event-driven computation offers a promising strategy to improve this efficiency by restricting activity to informative inputs and filtering out noise rather than maintaining continuous, dense activation. Neuromorphic and event-based computation systems employ activity patterns that are designed to limit responsiveness to meaningful input. This provides an approach for implementing sparse coding in artificial neural networks. For example, spiking neural networks and memristive neuromorphic computing are both driven by sparse, event-driven computation (Davies et al., 2018; Frenkel et al., 2023; Qiao et al., 2024). Recent architectures further demonstrate that sparse activation can enhance not only energetic efficiency, but also model performance (Zhao et al., 2024; Fruengel and Oberlaender, 2025; Fedus et al., 2022). ResNet spiking transformers and Liquid Networks, for instance, show improved generalization and robustness during sparse, adaptive computation (Hasani et al., 2021; Shrestha et al., 2024). Taken together, networks with sparse coding, potentially guided by dendritic computation and localized nonlinear activation, offer a blueprint for efficient next-generation artificial intelligence models that compute selectively, engaging resources only when and where they are needed.

## 7 From local computation to global coordination

While local dendritic error signals provide a mechanism for assigning credit at the level of individual synapses (i.e., “localizing credit”), a central question remains: how are these signals effectively propagated through a deep, multi-layered network? In particular, how can a local error signal arising in a late layer influence the synapses in earlier layers? For effective learning, information about errors in higher layers needs to, at least indirectly, guide plasticity throughout the network. In artificial neural networks, backpropagation addresses this problem through the global error propagation, allowing deep-layer errors to systematically update earlier layers. However, it remains unclear how networks relying solely on local learning rules can achieve comparable performance on hierarchical tasks. Recent work on target propagation, feedback alignment, and local learning in spiking networks may present alternative approaches but they have yet to match the performance of backpropagation on benchmarks such as ImageNet classification and large-scale language modelling (Nøkland, 2016; Lillicrap et al., 2016; Zenke et al., 2017). Insights from neuroscience suggest a two-part solution. First, dendritic spikes trigger local plasticity, effectively determining where network credit can be assigned within the network. Second, diffusely released neuromodulators (for example, dopamine for reward/error or acetylcholine for attention/novelty) provide global signals that determine when and how strongly credit should be assigned across the network. Together, these local and global mechanisms may enable coordinated credit assignment across distributed, hierarchical systems.

Theoretical advances in equilibrium-based learning provide a machine learning framework for integrating local and global signals. Approaches such as Equilibrium Propagation (Scellier and Bengio, 2017) demonstrate how, in energy-based models, a local error signal can emerge from the difference between two phases of network activity: a ‘free’ phase driven by model input and a ‘nudged’ phase influenced by target information. This is reminiscent of different dendritic (apical and basal) compartment integration in pyramidal neurons. If dendritic compartments can maintain local activity states, then mismatches between bottom-up sensory input (free phase) and top-down contextual input (nudged phase) could generate local error signals within a single neuron itself. This alternation between a data-driven and a target-driven phase is reminiscent of the wake and sleep phases used to train Boltzmann machines (Hinton and Sejnowski, 1986). Here, biological neural networks may represent a method to achieve an outcome functionally analogous to gradient descent, without relying on global error signals.

Future artificial intelligence systems could build on this principle by integrating local dendritic-like error signals with global neuromodulator-like signals. Similar mechanisms already exist in reinforcement learning (global reward signals) and attention-based architectures (global gating) (Vaswani et al., 2017; Barto, 2021), however, more explicit biologically inspired implementations remain largely unexplored. Developing explicit rules for combining the local plasticity signals with global reinforcement feedback could offer a novel biologically inspired mechanism for solving this integration problem.

## 8 Conclusion

Since Santiago Ramón y Cajal first reconstructed neurons, neuroscience has continued to magnify the smaller compartments within a single neuron, discovering remarkable computational capabilities. Alongside this, artificial intelligence has also continued to improve, with today's state-of-the-art modern large language models having exceeded all expectations with their abilities that were once thought unattainable. While both fields have grown in parallel, their histories highlight the value of cross-disciplinary exchange, where insights from biology can inform new computational paradigms.

This review has focused on recent findings showing that dendrites in pyramidal neurons are not passive structures but are active nonlinear processors. Through the generation of local spikes, dendrites can act as sequence predictors, coincidence detectors, and logic gates. Dendritic computation enables single pyramidal neurons to function as a multilayered, context-aware subnetwork which, in turn, contributes to sparse and efficient representations at the network level. We have presented artificial intelligence approaches that draw inspiration from these principles, demonstrating both their feasibility and potential advantages. Shifting from static, dense, and global architectures towards architectures that are adaptive, sparse, and local, may help address persistent challenges that remain in the field, such as learning and energy efficiency (Table 1). Dendrites therefore offer a compelling source of inspiration for artificial computation because, as we have demonstrated, they integrate nonlinear computation, local learning, structural plasticity, and sparse processing within a single, highly adaptable computational substrate, suggesting new architectural strategies for addressing credit assignment, continual learning, and energy-efficient computation in AI.

**Table 1:** **Dendritic computational principles and their proposed applications to artificial intelligence.** Summary of the four key concepts discussed in this review: contextual intelligence, local learning, continual learning, and energy efficiency. Each corresponds to pyramidal neuron dendrites and its potential relevance to artificial neural network design.

Contextual Intelligence	The two-compartment model of the pyramidal neuron provides a template for artificial intelligence that separates data from context, enabling distributed representations and parallel processing of information streams.
Local Learning	The ability of dendrites to generate local error signals provides a biologically plausible alternative to global backpropagation, potentially unlocking efficient learning in distributed systems.
Continual Learning	The structural remodeling of the dendritic tree creates a dynamic network topology, growing and pruning connections, helping solve catastrophic forgetting.
Energy Efficiency	The sparse, event-driven nature of dendritic spiking offers an alternate approach from energetically expensive dense matrix multiplication.

We compared representative dendrite-inspired models with state-of-the-art deep learning architecture to provide an overview of differences in computational principles, architecture, and reported performance (Table 2). This comparison illustrates a proof of concept of how even in their nascent form, dendrite-inspired approaches can achieve competitive performance on certain tasks by adopting some of the principles discussed in this review. Importantly, there remains significant room to innovate on this front, with significant potential computational and energetic benefits that could be achieved by implementing these strategies on neuromorphic hardware. Systematic benchmarking across tasks and hardware platforms is currently limited and remains an important direction for future work.

**Table 2:** **Comparison of dendritic-inspired models against State-of-the-Art (SOTA) deep learning.** Comparing architectural features and performance of current dendritic-inspired models with SOTA deep learning models (HTM: (Hawkins and Ahmad, 2016); ViTs: (Chen et al., 2025b); DNM: (Cerretti et al., 2025); Deep Learning: (Brown et al., 2020; He et al., 2016).

Model	Main Feature	Working Principle	Performance
Hierarchical Temporal Memory (HTM)	Active predictions: Side branches anticipate patterns based on past context.	Learn by timing and matching overlapping patterns without backpropagation (local learning and temporal integration)	Competitive performance to SOTA models at handling anomaly detection and noisy data; less effective at text generation or image tasks.
Bio-Probes & Vision Systems (e.g., AVS, HCdM)	On-off channels: Mimics the eye's physical wiring for motion and contrast.	Use built-in structural shortcuts instead of brute-force training.	Matches ViTs on motion classification while using substantially less (90%) training data.
Dendritic Network Models (DNM)	Sparse layout: Mimics the spread-out branch shapes of real biological networks.	Network becomes sparser and prunes itself dynamically during training.	Competitive performance with highly sparse activations, significantly reducing computational/memory requirements.
SOTA Deep Learning (e.g., GPT, ResNet)	Point neurons: Nodes as single computational units without dendrites	Dense, globally coordinated optimisation using backpropagation and large-scale parameter updates.	Outperforms across large-scale vision/language benchmarks, but requires massive amounts of power, data, and memory.

The path forward is not without challenges. Translating the slow and complex dynamics of biological growth into efficient algorithms that natively support sparse dendritic computation remains a formidable task. However, the continued interplay of neuroscience and artificial intelligence provides a promising path forward. By incorporating the principles of dendritic computation, we move a step closer to building artificial systems that better capture the tremendous efficiency, adaptability, and depth of the brain.

## Atuhor contributions

YH, NSG and LMP conceptualized the manuscript, contributed to Figure formation and manuscript writing.

## Declaration of competing interest

The authors declare that they have no known competing financial interests or personal relationships that could have appeared to influence the work reported in this paper.

## Data Availability

No data was used for the research described in the article.
